# Nephron Number, Single-Nephron Workload, and Podometrics as Predictors of Renal Function After Nephrectomy

**DOI:** 10.1016/j.ekir.2026.107099

**Published:** 2026-09-23

**Authors:** Christopher Paschen, Maximilian C. Wakonig-Koeller, Juliane Hennenberg, Johannes Kläger, Maja Nackenhorst, Andre Oszwald, Michael Kammer, Menno Pruijm, Nicolas Kozakowski, Dietmar Tamandl, Heinz Regele, Rainer Oberbauer

**Affiliations:** 1 Division of Nephrology and Dialysis, Department of Medicine III, Medical University of Vienna, Vienna, Austria; 2 Department of Pathology, Medical University of Vienna, Vienna, Austria; 3 Department of Biomedical Imaging and Image-Guided Therapy, Medical University of Vienna, Vienna, Austria; 4 Center for Medical Data Science, Institute of Clinical Biometrics, Medical University of Vienna, Vienna, Austria; 5 Department of Medicine, Service of Nephrology and Hypertension, University Hospital of Lausanne and University of Lausanne, Lausanne, Switzerland

**Keywords:** deep learning, kidney morphometry, nephrectomy, nephron number, podometrics, single-nephron eGFR

## Abstract

**Introduction:**

Nephrectomy results in abrupt nephron loss and variability in postoperative estimated glomerular filtration rate (eGFR) trajectories that are incompletely explained by baseline clinical factors. Adaptive hyperfiltration has been hypothesized to increase single-nephron workload, with potential consequences for podocyte structure. However, nephron number, single-nephron filtration, and podocyte structural characteristics have rarely been examined jointly as determinants of functional adaptation after nephron loss. We therefore evaluated their association with eGFR compensation after uninephrectomy.

**Methods:**

We retrospectively assessed nephron number, single-nephron filtration, and podometrics in patients undergoing nephrectomy for renal tumors, using imaging and stereology on nonneoplastic tissue. Renal compensation (ΔeGFR) was calculated at 1 year postsurgery, and structural-functional parameters were compared across ΔeGFR tertiles using analysis of variance.

**Results:**

A total of 54 patients (mean age: 67 ± 13 years; 43% female) were stratified into ΔeGFR tertiles. Presurgical baseline eGFR was 84 ± 15 ml/min per 1.73 m^2^ and was similar across tertiles. Patients with the poorest compensation (most negative ΔeGFR) exhibited lower nephron number (0.81 ± 0.20 × 10^6^ vs. 1.12 ± 0.39 and 1.20 ± 0.42 × 10^6^ in the greater and intermediate compensation groups; *P* = 0.005), glomerular hypertrophy (3.0 ± 0.7 × 10^6^ μm^3^ vs. 2.2 ± 0.5 and 2.5 ± 0.6 × 10^6^ μm^3^ in the greater and intermediate compensation groups; *P* = 0.004), and higher single-nephron filtration (54 ± 15 nl/min per 1.73 m^2^ vs. 42 ± 13 and 39 ± 13 nl/min per 1.73 m^2^ in the greater and intermediate compensation groups; *P* = 0.005) preoperatively. Podocyte-specific metrics were similar across tertiles.

**Conclusion:**

Together, these findings frame compensatory adaptation after nephrectomy as a function of nephron endowment, structural changes, and single-nephron workload, extending beyond conventional clinical risk profiles alone. By integrating parameters rarely examined together, this study provides a comprehensive view of post-nephrectomy functional adaptation.

## Graphical abstract

**Figure:**
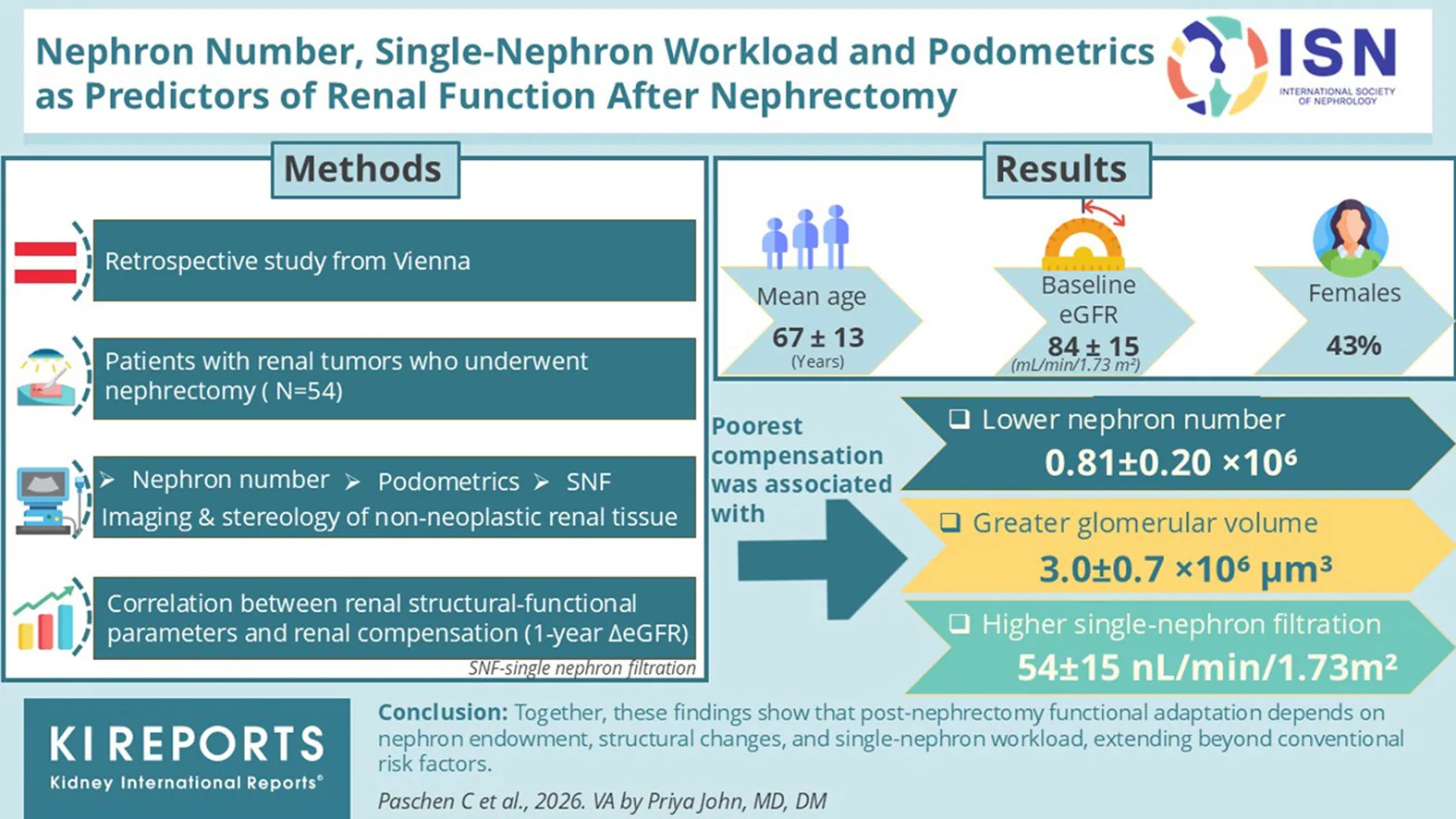

Nephrectomy, performed both in the context of living kidney donation and for oncologic indications, is followed by marked interindividual variability in postoperative eGFR trajectories.^1^^,^^2^ Living donors undergo nephrectomy in the absence of overt kidney disease with an expected postoperative course similar to the general population.^3^^,^^4^ In contrast, patients undergoing oncologic nephrectomy exhibit greater comorbidity and structural heterogeneity of the nonneoplastic kidney parenchyma.^5^ Despite these differences, substantial overlap exists in postoperative functional outcomes across both settings, with a subset of individuals experiencing a disproportionate decline in eGFR and progression toward chronic kidney disease.^1^^,^^2^^,^^6^, ^7^, ^8^, ^9^, ^10^, ^11^, ^12^ Established risk factors, including baseline kidney function and comorbidities, explain this variability incompletely.^1^^,^^2^^,^^6^, ^7^, ^8^, ^9^, ^10^, ^11^, ^12^

At a fundamental level, nephrectomy entails an abrupt loss of functional renal parenchyma and therefore provides a unique human model to study how nephron endowment, glomerular structure, and single-nephron function jointly to determine the renal functional capacity and reserve.^1^^,^^13^, ^14^, ^15^ Nephron number represents a key quantitative determinant of this adaptive capacity, because the functional workload per nephron rises in proportion to nephron loss after nephrectomy.^5^^,^^7^^,^^9^^,^^16^

Beyond nephron quantity, structural properties of glomeruli are recognized as critical modifiers of compensatory filtration capacity after nephron loss.^7^^,^^10^^,^^12^^,^^13^ Among these, podocytes play a pivotal role as terminally differentiated cells with limited regenerative potential, representing a rate-limiting component of the glomerular filtration barrier.^15^ In the setting of increased single-nephron filtration demand, compensatory glomerular hypertrophy may increase biomechanical demands on podocytes, requiring enlargement to maintain coverage of the expanding capillary surface.^13^^,^^15^ Structural imbalance between glomerular size and podocyte adaptive capacity has been associated with glomerular injury, podocyte depletion, and functional impairment after nephrectomy.^6^, ^7^, ^8^, ^9^, ^10^, ^11^, ^12^^,^^17^^,^^18^

Although podometrics has been associated with adverse renal outcomes across diverse disease states, evidence remains fragmented, because most studies examined podocyte characteristics in isolation from single-nephron filtration and vice versa.^6^^,^^7^^,^^9^, ^10^, ^11^, ^12^^,^^17^, ^18^, ^19^, ^20^ In the nephrectomy setting, studies have either described long-term eGFR trajectories based on clinical characteristics without structural assessment, or assessed nephron number using predominantly dichotomous outcomes.^1^^,^^2^^,^^6^, ^7^, ^8^, ^9^, ^10^, ^11^, ^12^ Podometric analyses have likewise been limited to dichotomous chronic kidney disease outcomes or short-term follow-up.^8^^,^^17^^,^^18^ Accordingly, the combined contribution of nephron quantity and podocyte structural resilience to postnephrectomy functional adaptation remains poorly defined.

We therefore hypothesized that an integrative assessment of nephron number, single-nephron filtration, and podocyte structural characteristics would better explain interindividual variability in compensatory functional adaptation after nephrectomy. To test this hypothesis, we quantified nephron number and single-nephron filtration alongside glomerular morphometry and podometrics in an oncologic nephrectomy cohort and examined their associations with the relative change in eGFR from baseline to 1-year follow-up. This integrative approach aims to elucidate key structural and functional determinants of renal functional compensation after nephron loss.

## Methods

### Population

This study included patients with a renal tumor who had a nephrectomy at the Medical University of Vienna between 2013 and 2018. Patients were eligible if their urine dipstick test was negative for proteinuria, a tissue block of unaffected kidney tissue from the nephrectomy was available, and the preoperative staging computed tomography scan encompassed the entire contralateral kidney. Patients were excluded if they underwent partial nephrectomy, a second nephrectomy for tumor recurrence, or received neoadjuvant chemotherapy or immunotherapy. The presence of arterial hypertension and diabetes mellitus was extracted from medical records. A study flow chart is presented in Figure 1, and a STROBE checklist is provided in Supplementary Table S1. The study protocol was approved by the institutional review board of the Medical University of Vienna (EK: 2107/2021) and conducted in accordance with the Declaration of Helsinki.

**Figure 1:**
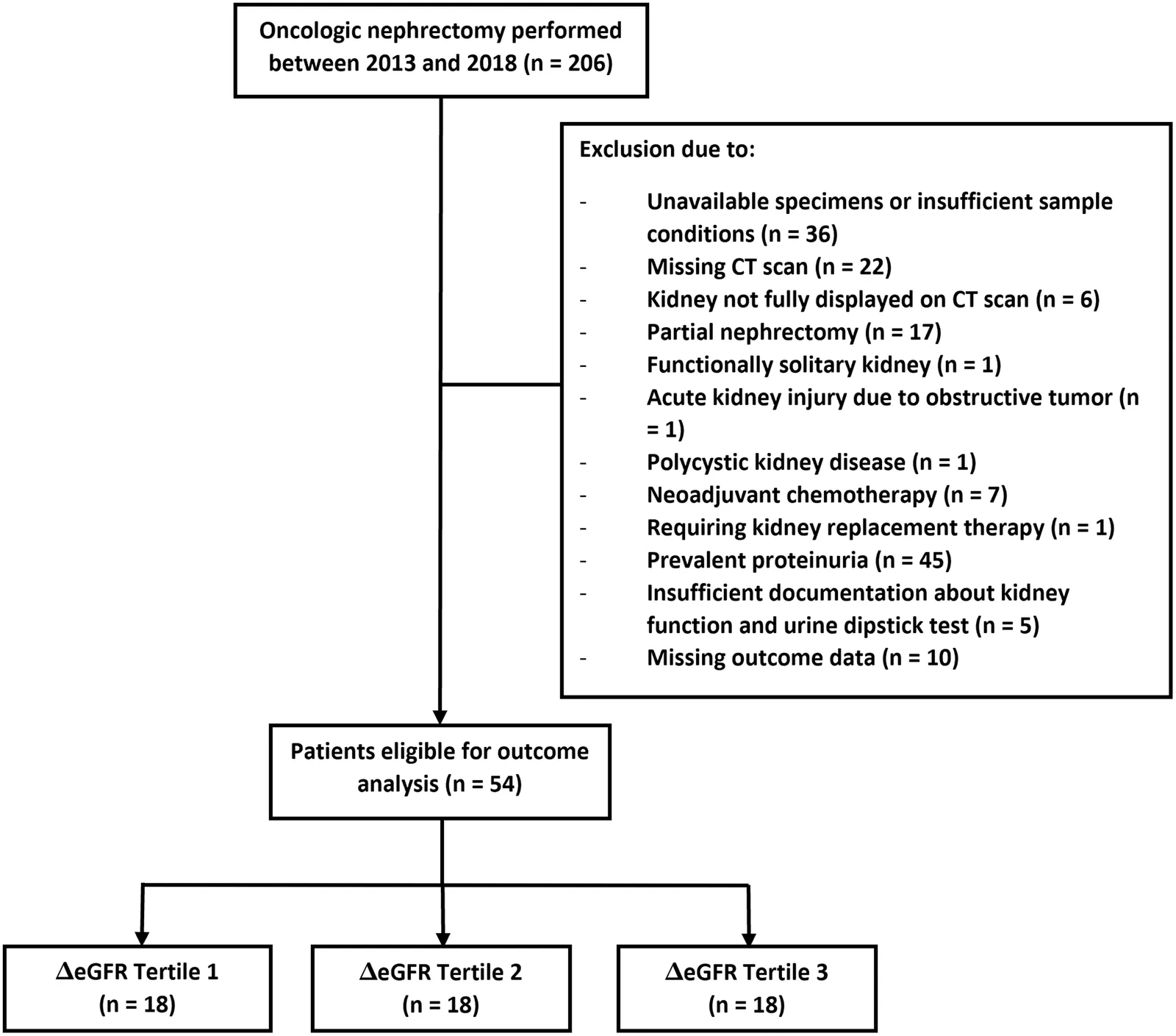
Study flow of individuals who underwent an oncologic nephrectomy for a renal tumor. Tertiles 1–3 represent greater, intermediate, and limited compensatory adaptation of the retained kidney after nephrectomy, respectively, based on the relative change in eGFR from baseline to 1-year follow-up (ΔeGFR). Median (interquartile range) ΔeGFR values were –18% (–21% to –6%), –33% (–35% to –28%), and –48% (–54% to –44%) for tertiles 1, 2, and 3, respectively. CT, computed tomography; eGFR, estimated glomerular filtration rate.

### Sample acquisition, staining, and routine diagnostics

All kidney specimens were obtained as wedge biopsies from unaffected parts of tumor nephrectomies. Specimens underwent histopathologic evaluation by an expert renal pathologist (H.R.) for mesangial expansion, intimal fibrosis, interstitial fibrosis, tubular atrophy, and arteriolar hyalinosis, scored semiquantitatively as absent, mild (°I), moderate (°II), or severe (°III) lesions.

After routine histopathologic assessment, paraffin-embedded sections were processed for immunohistochemical staining. Sections were deparaffinized using a xylene substitute (Neo-Clear, Sigma-Aldrich/Merck KGaA, Darmstadt, Germany) and rehydrated through graded ethanol, and subjected to antigen retrieval in citrate buffer (pH 6.0) by autoclaving for 20 minutes; 3% hydrogen peroxide was applied to block endogenous peroxidase activity. Podocyte nuclei were stained by immunohistochemistry using a Wilms tumor 1 protein (WT1)-specific antibody (abcam 89901, Cambridge, UK; 1:500 dilution) and incubated overnight at 4 °C. A polymer-based detection system served as the secondary antibody (Epredia Lab Vision UltraVision LP Detection System; Fremont, CA). Diaminobenzidine (DAB, Thermo Scientific Metal Enhanced DAB Substrate Kit 34065; Rockford, IL) was used for 2 minutes. Slides were counterstained with hemalum, mounted using Eukitt (O. Kindler GmbH, Freiburg, Germany) and digitized using a 3D Histech Pannoramic 250 slide scanner.

### Nephron number estimation

Morphometrical analyses were performed using QuPath (v0.3.2).^21^ Nephron number and single-nephron parameters were estimated as described by Denic *et al.*^5^ by combining histologic information from unaffected tissue of the resected kidney with preoperative computed tomography-scan-assessed cortical volume of the remaining kidney. Nonsclerotic glomeruli (NSG) were preannotated by a deep-learning-based segmentation algorithm and manually refined.^17^^,^^22^ Cortex area was delineated manually (Figure 2). NSG density estimation corrected for missing glomerular tufts in Bowman spaces, with entire Bowman capsules without a glomerular tuft and partial capsules at the specimen margin included, and partial NSG tufts at wedge edges traced and incorporated, was conducted using the following equation^5^:

$$\text{Corrected}\;\text{NSG}\;\text{volumetric}\;\text{density}\;\left(\text{per}\;{mm}^{3}\;\text{of}\;\text{cortex}\right)=\left(\frac{1}{1.382}\right)\times \sqrt{\frac{{\left(\frac{Total\;No.\;of\;NSG}{Cortex\;Area}\right)}^{3}}{\frac{NSG\;area+missing\;NSG\;No.\times Mean\;NSG\;area}{Cortex\;Area}}}$$

where:

TotalNo.ofNSG=No.ofcompletetracedandmissingNSG+0.675×No.ofpartialtracedandmissingNSG

**Figure 2:**
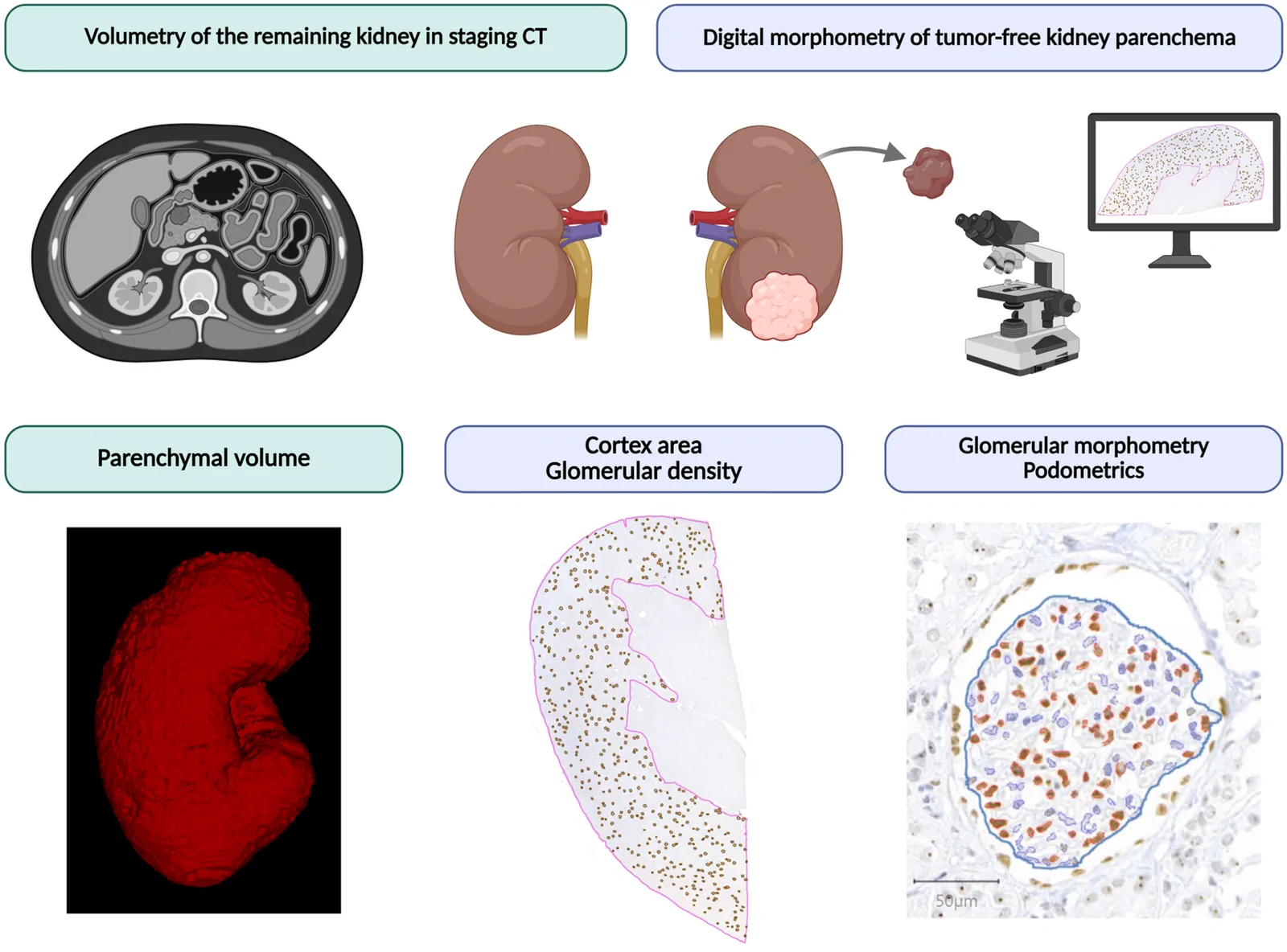
Overview of the morphometry workflow. Parenchymal volumetry of the remaining kidney derived from staging computed tomography scans for nephron number estimation (green). Histologic characterization of tumor-free kidney tissue in nephrectomy specimens (blue). Estimation of glomerular density on immunohistochemistry-stained sections using a nuclear WT1 antibody. Glomerulus segmentation, restricted to the glomerular tuft and excluding Bowman space and capsule. Podocyte nucleus detection confined to the glomerular tuft profile and excluding parietal epithelial cells. Created in BioRender. Paschen, C. (2026) https://BioRender.com/ym2m10u. CT, computed tomography, WT1, Wilms tumor 1.

Parenchymal volume in presurgical staging CT scans was measured using Horos (version 3.3.6, Figure 2). Cortical volume was ascertained using the equation for cortical volume in tumorpatients^5^:

$$\text{Cortical}\;\text{volume}\;\left({mm}^{3}\right)=0.74\times \text{Kidney}\;\text{parenchymal}\;\text{volume}-5500\;-2400\;\left(\text{if}\;\text{slice}\;\text{thickness}=5\text{mm}\right)-23400\;\;(\text{if}\;\text{slice}\;\text{thickness}=6-10\;\text{mm})$$

The nephron number of the remaining kidney was estimated using the derived cortical volume, corrected NSG density, and the reported correction factors for volumetric shrinkage (1.365) and the loss of tissue perfusion pressure (1.366)^5^:

$$\text{Nephron}\;\text{number}=\frac{\text{Cortex}\;\text{volume}\;\text{of}\;\text{retained}\;\text{kidney}\times \text{NSG}\;\text{density}}{1.365\;x\;1.366}$$

Single-nephron eGFR was calculated by dividing eGFR by nephron number, with eGFR determined using the Chronic Kidney Disease Epidemiology Collaboration (CKD-EPI) 2021 creatinine equation.^5^^,^^23^

$$\text{Single}-\text{nephron}\;\text{eGFR}=\frac{\text{eGFR}}{\text{Nephron}\;\text{number}}$$

### Modeling of intraglomerular pressure

Glomerular capillary pressure ($P_{GC}$) was estimated by rearranging the Starling equation for glomerular ultrafiltration:

$$P_{GC}=\frac{J_{V}}{K_{f}}{+P}_{BS}+\pi_{GC}$$

where _*JV*_ is the single-nephron eGFR, *K*_*f*_ the ultrafiltration coefficient (simplified as $K_{f}=V_{\text{Glom}}$, because hydraulic permeability ($L_{p}$) and a scaling factor for capillary surface area ($f_{cap}$) could not be directly measured in our specimens), *P*_*BS*_ Bowman space pressure (assumed to be 12 mmHg), and π_GC_ plasma oncotic pressure within glomerular capillaries, calculated from serum protein using the Landis-Pappenheimer equation:

$$\pi_{GC}\left(\text{mmHg}\right)\approx 2.1\times \left[\text{Protein}\;\left(g/\text{dl}\right)\right]+0.16\times {\left[\text{Protein}\;\left(g/\text{dl}\right)\right]}^{2}+0.009\times {\left[\text{Protein}\;\left(g/\text{dl}\right)\right]}^{3}$$

### Stereological evaluation of glomerular volume and podometrics

Glomerular volume was estimated from the mean cross-sectional area of traced nonsclerotic glomerular tuft profiles according to the stereological method of Weibel and Gomez (β: 1.38; size distribution coefficient: 1.01)^24^^,^^25^:

$$Glomerular\;volume={Mean\;glomerular\;profile\;area}^{1.5}\times \frac{1.38}{1.01}$$

WT1-positive podocyte nuclei were identified by positive cell detection on segmented glomerular tufts (Figure 2).^21^ Podocyte density was estimated using the stereological single- section method.^26^ The apparent nuclear caliper diameter *d* was measured from WT1-positive nuclear polygons.^17^^,^^18^^,^^26^ The section thickness (T) was measured individually for each specimen via the Z-axis distance between the uppermost and lowermost in-focus planes on a Zeiss Axio Imager 2 using a 100x oil objective; (cohort mean: 4.54 ± 0.23 μm).^17^^,^^18^ *d* was then corrected using a quadratic formula to obtain the true nuclear diameter (*D*), assuming a podocyte nuclear shape coefficient (k) of 0.72^17^^,^^18^^,^^26^:

$$D=\frac{d-T+\sqrt{{\left(d-T\right)}^{2}+4kdT}}{2k}$$

Podocyte density estimation necessitates a correction factor (CF) for the detected podocyte nuclei on a histologic section^26^:

$$CF={\left(\frac{D}{T}+1\right)}^{-1}$$

Podocyte density was calculated using^26^:

$$Podocyte\;Density=\frac{Detected\;podocyte\;nuclei\times CF}{T\times Total\;tuft\;area\;per\;slide}$$

Multiplying podocyte density and glomerular volume estimated the podocyte number per glomerular tuft. Single-podocyte eGFR was defined as the ratio of single-nephron eGFR to podocyte number per tuft.^27^ Podocyte nuclear volume was calculated stereologically^17^^,^^18^^,^^20^:

$$Podocyte\;nucleus\;volume=\frac{4\pi }{3}\times {\left(\frac{2}{\pi }\times d\right)}^{3}$$

### Statistical analysis

Metric variables are presented as mean ± SD for normally distributed data, and median with interquartile range for skewed data. Distributional assumptions were evaluated graphically. Groups were defined based on tertiles of functional compensation (ΔeGFR), calculated as the relative change from the baseline eGFR (prenephrectomy) to 1-year follow-up eGFR. Intergroup comparisons of metric variables were performed using one-way analysis of variance with Tukey *post hoc* test; homogeneity of variances was confirmed by Levene’s test. Sensitivity analyses were performed using analysis of covariance with Bonferroni-adjusted *post hoc* comparisons, adjusting for diabetes and visceral obesity (defined by a dichotomous threshold of visceral adipose tissue area >100 cm^2^ at L3)^18^ as a fixed factor. Distributional assumptions of residuals were evaluated using histograms and Q-Q-plots. In sensitivity analyses, deindexed single-nephron eGFR (single-nephron eGFR × [BSA/1.73 m^2^]) was additionally evaluated using analysis of variance and analysis of covariance. Semiquantitative histopathologic scores were compared using the Kruskal-Wallis test, and categorical variables were compared using the chi-square test.

Two-sided ***P*** values < 0.05 were considered statistically significant. No adjustments for multiple comparisons were performed. Statistical analyses were conducted using SPSS (version 21; IBM Corp., Armonk, NY, USA) and GraphPad Prism (version 8; GraphPad Software, La Jolla, CA, USA).

## Results

### Patient Characteristics

This study included 54 patients who underwent nephrectomy for renal tumors. Mean ΔeGFR values at 1 year were –16±8, –32±4, and -50±8 ml/min per 1.73 m^2^ for tertiles 1–3, representing greater, intermediate, and limited compensatory adaptation, respectively. Baseline characteristics before nephrectomy did not differ between groups, except for type 2 diabetes and visceral obesity (Table 1). The mean prenephrectomy eGFR was 84±14 ml/min per 1.73 m^2^. Overall, 43% of patients were female, and the average age was 67±13 years. Hypertension was present in 69% of patients. Diabetes was observed in 28% of the cohort and was most frequent in the tertile with limited compensation (56%; *P* = 0.005). BMI, waist circumference, and visceral adipose tissue area did not differ significantly; however, visceral obesity was more prevalent in tertiles 2 and 3 (89% and 78% vs. 50% in tertile 1, ***P*** = 0.03). There were no differences in posterior and lateral perirenal adipose tissue or perirenal fat stranding.

**Table 1:** Baseline demographics depending on tertiles of relative ΔeGFR at 1-year follow-up

Characteristic	Tertile 1 (n=18)	Tertile 2 (n=18)	Tertile 3 (n=18)	*P*
Sex (female), no. (%)	8 (44%)	7 (39%)	8 (44%)	0.93
Age, yr	65 (±14)	67 (±12)	69 (±11)	0.62
eGFR, ml/min per 1.73 m^2^	86 (±15)	86 (±12)	80 (±16)	0.44
ΔeGFR, %	–16 (±8)	–32 (±4)	–50 (±8)	<0.001
Deindexed eGFR, ml/min	93 (±19)	99 (±17)	91 (±22)	0.39
Arterial hypertension, no. (%)	10 (56%)	14 (78%)	13 (72%)	0.33
Diabetes mellitus, no. (%)	3 (17%)	2 (11%)	10 (56%)	0.005^a^
Weight, kg	73.9 (±15.4)	83.0 (±10.3)	80.1 (±19.1)	0.20
Height, m	1.70 (±0.11)	1.72 (±0.07)	1.70 (±0.10)	0.71
BSA, m^2^	1.8 (±0.3)	1.9 (±0.2)	2.0 (±0.2)	0.21
BMI, kg/m^2^	25.5 (±4.2)	28.0 (±3.2)	27.6 (±5.9)	0.22
Waist circumference, cm	97 (±14)	103 (±7)	102 (±14)	0.27^b^
Visceral adipose tissue, cm^2^	154 (±129)	193 (±83)	177 (±110)	0.55
Visceral obesity, no. (%)	9 (50%)	16 (89%)	14 (78%)	0.03^a^
Posterior perirenal adipose tissue, mm	11 (±11)	14 (±8)	12 (±10)	0.55
Lateral perirenal adipose tissue, mm	15 (±10)	19 (±11)	18 (±8)	0.43
Perirenal fat stranding, no. (%)	10 (56%)	9 (50%)	9 (50%)	0.83
Serum protein, g/dl	7.0 (±0.7)	7.1 (±0.6)	6.8 (±0.6)	0.52
Cholesterol, mg/dl	178 (±53)	187 (±5 2)	193 (±45)	0.68
HDL, mg/dl	57 (±23)	52 (±14)	50 (±16)	0.53
LDL, mg/dl	102 (±42)	110 (±46)	114 (±37)	0.70
Triglycerides, mg/dl	121 (±69)	135 (±63)	125 (±48)	0.80
RAS inhibitors, no. (%)	6 (33%)	12 (67%)	10 (56%)	0.13
SGLT2 inhibitors, no. (%)	0 (0%)	1 (6%)	1 (6%)	0.57
Lipid-lowering drugs, no. (%)	7 (39%)	3 (17%)	5 (28%)	0.33

ANOVA, analysis of variance; BMI, body mass index; BSA, body surface area; eGFR, estimated glomerular filtration rate; HDL, high-density lipoprotein cholesterol; LDL, low-density lipoprotein cholesterol; RAS, renin-angiotensin system; SGLT2, sodium glucose co-transporter 2; VO, visceral obesity.

Tertiles 1–3 represent greater, intermediate, and limited compensatory adaptation of the retained kidney after nephrectomy, respectively, based on the relative change in eGFR from baseline to 1 year follow-up (ΔeGFR). Median (interquartile range) ΔeGFR values were –18% (–21% to –6%), –33% (–35% to –28%), and –48% (–54% to –44%) for tertiles 1, 2, and 3, respectively. Normally distributed variables are displayed as means and SDs. VO was defined as visceral adipose tissue area ≥ 100 cm^2^.

a *P* < 0.05 in ANOVA.

b Welch test performed.

### Routine histopathologic assessment

Histopathologic examination revealed chronic vascular and tubulointerstitial changes. Mild fibrointimal thickening (arteriosclerosis) was observed in 35% of patients, whereas 63% exhibited moderate-to-severe fibrointimal thickening. Arteriolar hyalinosis was present in 24% of the cohort. Interstitial fibrosis and tubular atrophy were each observed in 41% of patients, predominantly as mild lesions (Supplementary Table S2). All assessed histopathologic scores were similarly distributed across groups.

### Nephron number estimation

The kidney cortex volume of the remaining kidney was similar across compensation tertiles (*P* = 0.18). The mean cortical area of the wedge biopsy sections was 116±45 mm^2^, containing on average 300±121 nonsclerotic glomerular tuft profiles and a median of 13 (IQR: 4–19) Bowman capsules without a visible glomerular tuft. Metrics used for nephron number estimation and podocyte morphometry are summarized in Supplementary Table S3.

Nonsclerotic glomerular density was comparable in the greater and intermediate compensation tertiles (17.3±5.1 and 18.2±6.1 per mm^3^; Table 2) but numerically lower in the tertile with limited compensation (14.5±3.4 per mm^3^; *P* = 0.07). Accordingly, the estimated nephron number of the remaining kidney was lowest in the group with limited compensatory adaptation (0.81±0.20 ×10^6^; Figure 3), whereas similar values were observed in the greater and intermediate compensation groups (1.12±0.39 and 1.20±0.42×10^6^, *P* = 0.005).

**Table 2:** Kidney morphologic and histopathologic findings depending on tertiles of relative ΔeGFR at 1 year follow-up

Characteristic	Tertile 1 (n=18)	Tertile 2 (n=18)	Tertile 3 (n=18)	*P*
Kidney morphology				
Parenchymal volume, cm^3^	173 (±47)	176 (±37)	152 (±47)	0.21
Cortex volume, cm^3^	122 (±35)	125 (±27)	106 (±35)	0.18
Parenchymal volume per m body height, cm^3^/m	102 (±25)	102 (±21)	89 (±25)	0.19
Cortex volume per m body height, cm^3^/m	72 (±19)	72 (±15)	62 (±19)	0.15
Parenchymal volume per m^2^ BSA, cm^3^/m^2^	93 (±24)	88 (±18)	77 (±18)	0.08
Cortex volume per m^2^ BSA, cm^3^/m^2^	65 (±18)	62 (±14)	53 (±14)	0.06
Glomerular metrics				
No. of nephrons per kidney, ×10^6^	1.12 (±0.39)	1.20 (±0.42)	0.81 (±0.20)^a^^,^^b^	0.005^c^
Nonsclerotic glomerular density, per mm^3^	17.3 (±5.1)	18.2 (±6.1)	14.5 (±3.4)	0.07
Sclerotic glomeruli, %	2 (0–3)	1 (0–2)	1 (0–4)	0.96
Glomerular volume, ×10^6^ μm^3a^	2.2 (±0.5)	2.5 (±0.6)^a^	3.0 (±0.7)^a^	0.004^c^
Podometrics				
Podocyte count per glomerulus	464 (±174)	514 (±134)	565 (±201)	0.22
Podocyte density, per 10^6^ μm^3^	213 (±72)	214 (±57)	193 (±63)	0.56
Podocyte nuclear volume, μm^3^	211 (±35)	213 (±28)	230 (±32)	0.17
Nephro-podometrics				
Total glomerular volume per kidney, mm^3^	2360 (±1044)	2829 (± 903)	2282 (±1060)	0.21
Total podocyte count per kidney, ×10^6^	531 (±295)	614 (±230)	467 (±295)	0.29
Tubular metrics				
Tubular count per mm^2^ cortical area	198 (±33)	198 (±59)	173 (±31)	0.16
Mean tubular area per mm^2^ cortical area, μm^2^	4974 (±907)	4939 (±1088)	5453 (±818)	0.20
Filtration and hemodynamic indices				
Single-nephron eGFR, nl/min per 1.73 m^2^	42 (±13)	39 (±13)	54 (±15)^a^^,^^b^	0.005^c^
Deindexed single-nephron eGFR, nl/min	46 (±17)	45 (±14)	59 (±14)^a^^,^^b^	0.008^c^
Single-podocyte eGFR, pl/min per 1.73 m^2^	104 (±56)	80 (±34)	111 (±57)	0.17
Glomerular capillary pressure, mmHg	56 (± 6)	53 (±5)	56 (±7)	0.42
Oncotic pressure, mmHg	25.6 (±3.8)	26.3 (±3.4)	24.9 (±3.5)	0.52

BSA, body surface area; eGFR, estimated glomerular filtration rate.

Tertiles 1–3 represent greater, intermediate, and limited compensatory adaptation of the retained kidney after nephrectomy, respectively, based on the relative change in eGFR from baseline to 1 year follow-up (ΔeGFR). Median (interquartile range) ΔeGFR values were –18% (–21% to –6%), –33% (–35% to –28%), and –48% (–54% to –44%) for tertiles 1, 2, and 3, respectively. Gaussian-distributed parameters are displayed as means SDs, non-Gaussian-distributed data as medians and interquartile ranges.

a *P* < 0.05 in post hoc analysis versus tertile 1.

b *P* < 0.05 in post hoc analysis versus tertile 2.

c *P* < 0.05 in ANOVA.

**Figure 3:**
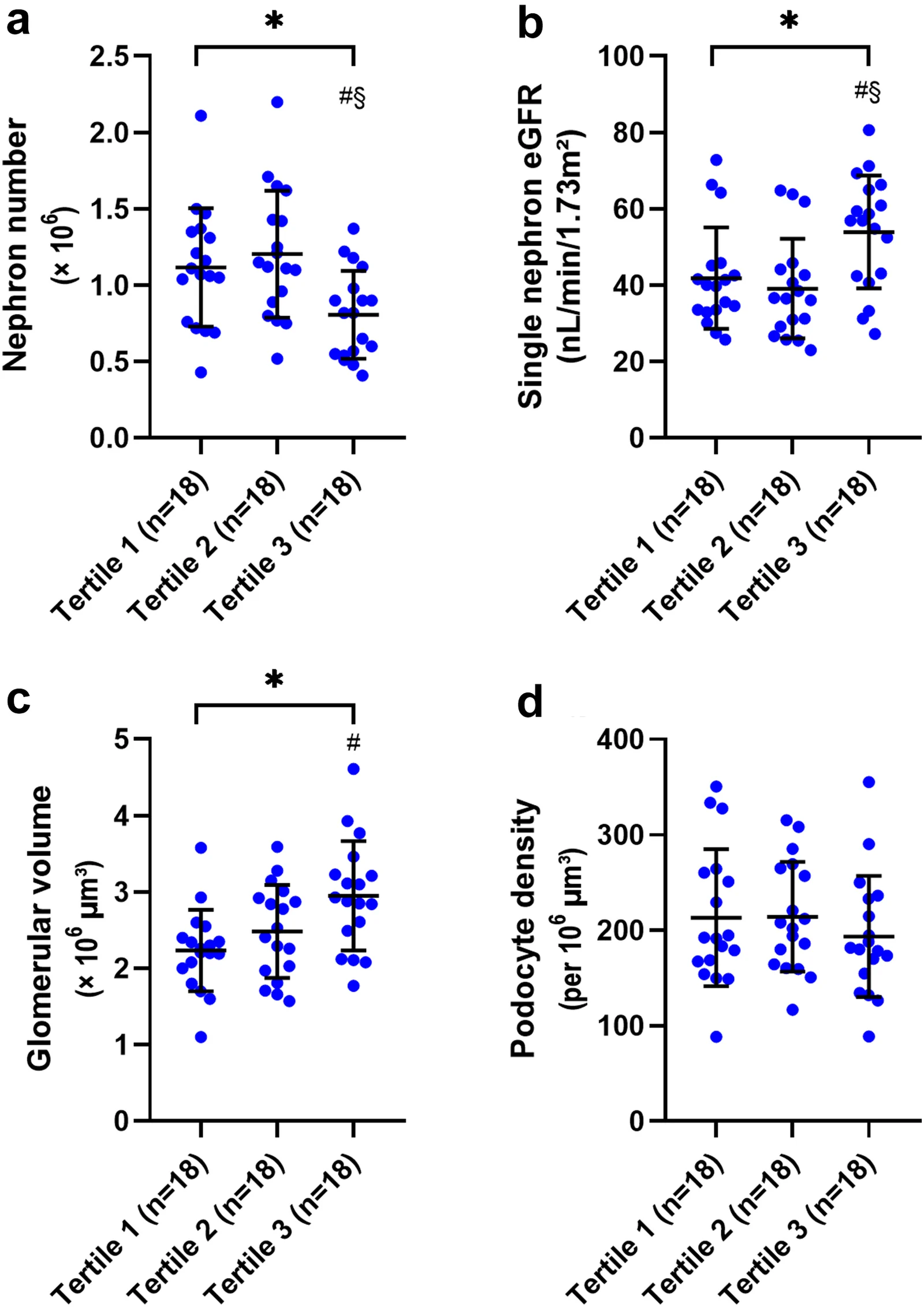
Structural-functional parameters stratified by ΔeGFR tertiles. ΔeGFR represents the relative change in eGFR from baseline to 1-year follow-up. Tertiles 1–3 correspond to greater, intermediate, and limited compensatory adaptation of the retained kidney after nephrectomy. Median (interquartile range) ΔeGFR values were –18% (–21% to –6%), –33% (–35% to –28%), and –48% (–54% to –44%) for tertiles 1, 2, and 3, respectively. (a) Nephron number, (b) single-nephron eGFR, (c) glomerular volume, and (d) podocyte density. Between-group comparisons were performed using analysis of variance with Tukey *post hoc* testing. ^∗^*P* < 0.05 in ANOVA, ^#^*P* < 0.05 versus tertile 1, ^§^*P* < 0.05 versus tertile 2. ANOVA, analysis of variance; eGFR, estimated glomerular filtration rate.

### Glomerular morphometry and podometrics

Mean glomerular volume increased progressively from the greater to the limited compensation group (2.2±0.5, 2.5±0.6, and 3.0±0.7 ×10^6^ μm^3^; *P* = 0.004; Figure 3). In contrast, podocyte count (464±174, 514±134, and 565±201, *P* = 0.22), podocyte density (213±72, 214±57, and 193±63 per 10^6^ μm^3^, *P* = 0.56), and podocyte nuclear volume (211±35, 213±28, and 230±32 μm^3^, *P* = 0.17) did not differ significantly across tertiles (Table 2). Total kidney glomerular volume and podocyte count were similar across groups (*P* = 0.21 and *P* = 0.29).

### Filtration indices and intraglomerular hemodynamics

Single-nephron eGFR was comparable in the greater and intermediate compensation tertiles but significantly higher in the group with limited compensatory adaptation (42±13, 39±13, and 54±15 nl/min per 1.73 m^2^, *P* = 0.005, Figure 3), indicating a higher filtration demand per nephron. Across the cohort, single-nephron eGFR was positively correlated with podocyte nuclear volume (*r*_p_: 0.44; *P* = 0.001, Figure 4A) and inversely with podocyte density (*r*_p_: –0.38; *P* = 0.005, Figure 4B). Although single-nephron eGFR was not associated with podocyte count per glomerulus (*r*_p_: –0.005; *P* = 0.97; Figure 4C), it showed a strong inverse correlation with total kidney podocyte count (*r*_p_: –0.62; *P* < 0.001; Figure 4D). Therefore, higher filtration demand per nephron is associated with podocyte nuclear hypertrophy and depletion (Figure 4).

**Figure 4:**
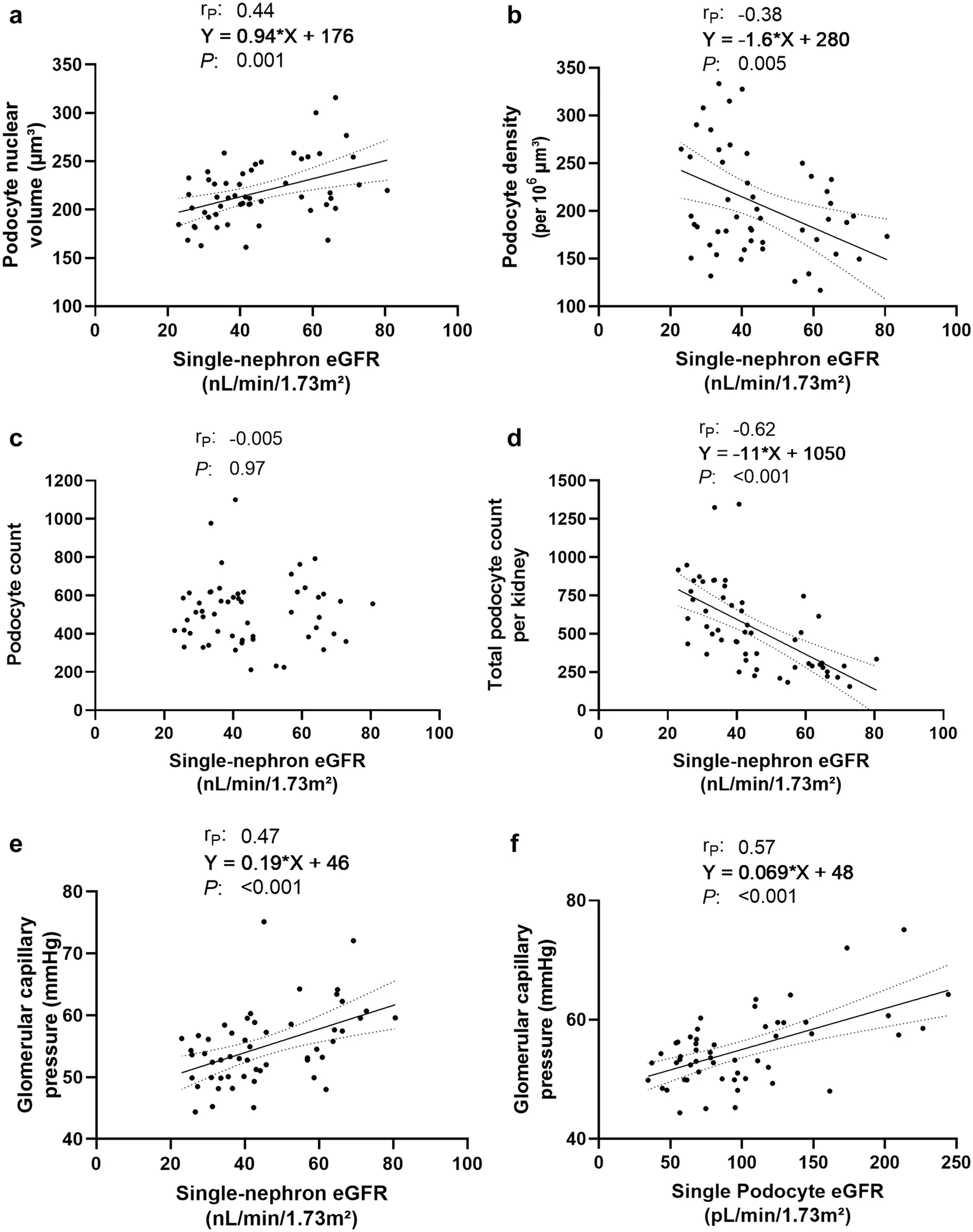
Correlations of single-nephron estimated glomerular filtration rate (eGFR) and structural-functional parameters. (a) Podocyte nuclear volume, (b) podocyte density, (c) podocyte count per glomerulus, and (d) per kidney, (e) glomerular capillary pressure. (f) Correlation of single-podocyte eGFR with glomerular capillary pressure.

Estimated glomerular capillary pressure did not differ across compensation tertiles (*P* = 0.42). By contrast, glomerular capillary pressure was strongly correlated with single-nephron eGFR (*r*_p_: 0.47; *P* < 0.001; Figure 4E) and single-podocyte eGFR (*r*_p_: 0.57; *P* < 0.001, Figure 4F).

### Sensitivity analysis

Intergroup differences for NSG density, nephron number, glomerular volume, and single-nephron eGFR remained significant after adjustment for diabetes and visceral obesity (Table 3). Because diabetes was more prevalent in the limited compensation tertile, morphologic parameters were additionally assessed according to diabetes status within this tertile and showed no significant differences (Supplementary Table S4). Further adjustment for age, baseline eGFR, and visceral obesity yielded consistent results (Supplementary Table S5).

**Table 3:** Sensitivity analysis of kidney morphologic and histopathologic parameters depending on tertiles of relative ΔeGFR at 1 year follow-up

Characteristic	Tertile 1 (*n* = 18)	Tertile 2 (*n* = 18)	Tertile 3 (*n* = 18)	*P*	*P* for DM	*P* for VO
	EMM (95% CI)	EMM (95% CI)	EMM (95% CI)			
Kidney morphology						
Parenchymal volume, cm^3^	202 (175–230)	174 (145–202)	148 (122–175)^a^	0.02^b^	0.051	0.96
Cortex volume, cm^3^	144 (123–164)	123 (101–144)	103 (83–123)^a^	0.02^b^	0.047^b^	0.98
Parenchymal volume per m body height, cm^3^/m	116 (101–131)	99 (83–115)	88 (73–103)^a^	0.03^b^	0.06	0.87
Cortex volume per m body height, cm^3^/m	83 (71–94)	70 (58–82)	61 (50–72)^a^	0.03^b^	0.053	0.87
Parenchymal volume per m^2^ BSA, cm^3^/m^2^	108 (95–121)	87 (74–101)	77 (65–89)^a^	0.004^b^	0.046^b^	0.34
Cortex volume per m^2^ BSA, cm^3^/m^2^	77 (67–87)	62 (51–72)	53 (44–63)^a^	0.004^b^	0.04^b^	0.36
Glomerular metrics						
No. of nephrons per kidney, ×10^6^	1.16 (0.90–1.42)	1.22 (0.95–1.49)	0.74 (0.49–0.99)^c^	0.02^b^	0.93	0.99
Nonsclerotic glomerular density, per mm^3^	15.6 (12.3–18.8)	18.7 (15.2–22.1)	13.6 (10.5–16.8)	0.28	0.12	0.72
Glomerular volume, ×10^6^ μm^3^	2.2 (1.7–2.6)	2.4 (2.0–2.8)	3.0 (2.6–3.4)^a^	0.02^b^	0.99	0.22
Podometrics						
Podocyte count per glomerulus	419 (296–541)	525 (399–652)	563 (445–681)	0.24	0.58	0.95
Podocyte density, per 10^6^ μm^3^	199 (154–243)	225 (179–272)	191 (148–234)	0.61	0.39	0.33
Podocyte nuclear volume, μm^3^	214 (193–235)	203 (181–225)	223 (203–243)	0.37	0.63	0.12
Nephro-podometrics						
Total glomerular volume per kidney, mm^3^	2370 (1676–3064)	2726 (2007–3446)	2110 (1441–2778)	0.57	0.99	0.28
Total podocyte count per kidney,× 10^6^	490 (295–684)	632 (430–834)	424 (237–612)	0.42	0.61	0.90
Tubular metrics						
Tubular count per mm^2^ cortical area	194 (165–224)	179 (149–210)	166 (138–195)	0.39	0.67	0.77
Mean tubular area per mm^2^ cortical area, μm^2^	5124 (4495–5752)	5236 (4584–5889)	5693 (5088–6299)	0.41	0.58	0.88
Filtration and hemodynamic indices						
Single-nephron eGFR, nl/min per 1.73 m^2^	38 (29–47)	38 (29–47)	61 (52–70)^a^^,^^c^	0.001^b^	0.87^b^	0.78
Deindexed single-nephron eGFR, nl/min	42 (33–52)	43 (33–53)	67 (57–76)^a^^,^^c^	0.001^b^	0.60	0.72
Single-podocyte eGFR, pl/min per 1.73 m^2^	100 (64–136)	74 (36–111)	119 (84–153)	0.25	0.97	0.76
Glomerular capillary pressure, mm Hg	54.1 (49.9–58.4)	53.5 (49.1–58.0)	56.8 (52.6–60.9)	0.59	0.64	0.34
Oncotic pressure, mm Hg	24.6 (22.3–27.0)	26.4 (24.0–28.9)	24.6 (22.3–26.9)	0.69	0.003^b^	0.48

ANCOVA, analysis of covariance; BSA, body surface area; CI, confidence intervals; DM, diabetes mellitus; EMM, estimated marginal means; eGFR, estimated glomerular filtration rate; VO, visceral obesity.

Tertiles 1–3 represent greater, intermediate, and limited compensatory adaptation of the retained kidney after nephrectomy, respectively, based on the relative change in eGFR from baseline to 1 year follow-up (ΔeGFR). Median (interquartile range) ΔeGFR values were –18% (–21% to –6%), –33% (–35% to –28%), and –48% (–54% to –44%) for tertiles 1, 2, and 3, respectively. Parameters are displayed as EMM and 95% CI.

a P < 0.05 in Bonferroni-adjusted post hoc analysis versus tertile 1.

b P < 0.05 in ANCOVA.

c P < 0.05 in Bonferroni-adjusted *post hoc* analysis versus tertile 2.

## Discussion

Our data show that impaired functional compensation 1 year after nephrectomy reflects a distinct structural-functional phenotype characterized by reduced nephron number, higher single-nephron filtration, and larger glomerular volume despite comparable baseline eGFR. These findings extend previous studies linking glomerular hypertrophy to progressive kidney disease after nephrectomy by demonstrating that structural-functional differences are already associated with altered early postnephrectomy compensation.^7^^,^^9^, ^10^, ^11^, ^12^

Patients with the strongest impairment of compensation exhibited lower nephron number despite comparable baseline eGFR, mainly driven by a reduced NSG density. This was accompanied by increased single-nephron eGFR, consistent with the concept of compensatory hyperfiltration at the single-nephron level.^13^ These findings suggest that reduced nephron endowment and an increased single-nephron workload at baseline may limit the capacity for further compensatory increases and thus constrain global eGFR recovery after nephrectomy.

Single-nephron filtration values in our study were roughly half of those reported by Denic *et al.*^16^ This difference can be largely explained by 2 methodological factors: the use of eGFR rather than iothalamate-measured GFR and our nephron number estimation method, which corrects for missing glomerular tufts and consequently yields higher nephron counts and lower calculated single-nephron eGFR.^5^^,^^28^, ^29^, ^30^, ^31^ Despite these lower absolute values, patients with reduced nephron number exhibited higher single-nephron filtration.

Sustained hyperfiltration is a well-recognized driver of glomerular hypertrophy and increased functional demand on podocytes, reflecting structural adaptation to increased single-nephron workload and potentially contributing to adverse renal outcomes.^13^^,^^16^^,^^28^^,^^32^, ^33^, ^34^ Consistent with these concepts, single-nephron eGFR correlated with podocyte nuclear volume and inversely with podocyte density, supporting an association between single-nephron workload and podocyte remodeling.^35^ However, the biological role of podocyte nuclear enlargement remains uncertain. It may reflect increased functional demand associated with higher single-nephron filtration but may also accompany age- and hypertension-associated arterionephrosclerotic remodeling.^36^ Arterionephrosclerosis represents a morphologic continuum from preserved or enlarged glomeruli through tuft contraction, podocyte depletion, capsular fibrosis, and loss of tubular continuity to focal global glomerulosclerosis.^36^ Podocyte nuclear enlargement may accompany adaptive podocyte remodeling in enlarged glomeruli but may also occur in contracting glomeruli with podocyte senescence and loss.^36^ Because classifying individual glomeruli according to tuft contraction, capsular fibrosis, or tubular continuity was beyond the scope of our study, we cannot distinguish between these potentially overlapping processes.

A recent study by Denic *et al.*^8^ reported that lower baseline podocyte number and density were associated with long-term kidney function decline after oncologic nephrectomy. Although podometric parameters did not differ across compensation tertiles within our cohort, the studies addressed different outcomes: Denic *et al.*^8^ evaluated long-term decline from the postnephrectomy baseline, whereas we examined early compensation from the presurgical baseline to 1 year after nephrectomy. The apparent difference may therefore reflect different outcome definitions, reference time points, follow-up duration, and cohort composition. However, most previous studies on podometrics did not assess single-nephron eGFR, leaving it unclear whether altered single-nephron filtration was present.^8^^,^^17^, ^18^, ^19^, ^20^^,^^37^, ^38^, ^39^

In our cohort, glomerular capillary pressure correlated with single-nephron eGFR, supporting the presence of increased single-nephron workload during hyperfiltration. However, no differences were observed across compensation tertiles, which may partly reflect limitations of our hemodynamic model, because parameters such as capillary surface area and hydraulic conductivity could not be individually estimated and were therefore treated as fixed values. Nevertheless, these findings are compatible with the concept that compensatory glomerular hypertrophy may preserve filtration efficiency and buffer increases in glomerular capillary pressure despite higher single-nephron filtration.^16^^,^^40^ Accordingly, our findings suggest structural adaptation in the setting of increased single-nephron filtration rather than providing evidence for overt glomerular hypertension.

In the context of recent concepts proposed by Haruhara and colleagues, total kidney glomerular volume, total kidney podocyte count, and single-podocyte eGFR were not independently associated with the relative change in eGFR.^27^ However, total kidney parameters correlated inversely with single-nephron eGFR, suggesting that lower aggregate glomerular and podocyte reserve was associated with greater filtration workload at the single-nephron level.

Patients with moderate and severe impairment had a greater prevalence of visceral obesity, and type 2 diabetes was more common among individuals with severely impaired compensation. Both elevated body weight and type 2 diabetes may contribute to glomerular alterations, increased filtration workload, and progressive kidney dysfunction.^16^, ^17^, ^18^^,^^24^^,^^28^^,^^34^^,^^35^^,^^37^^,^^38^^,^^41^^,^^42^ In contrast, arterial hypertension was similarly distributed across groups. Importantly, the reported structural-functional pattern remained robust in sensitivity analyses adjusted for visceral obesity and type 2 diabetes. Within the group with limited compensatory adaptation, patients with and without type 2 diabetes exhibited similar nephron endowment, structural changes, and single-nephron filtration. Thus, these comorbidities may contribute to, but do not fully explain, the observed structural-functional phenotype.

Although age did not differ significantly between groups, patients with limited compensatory adaptation were numerically older. This is relevant because nephron number decreases and glomerular volume increases with aging.^16^^,^^24^^,^^43^ The frequent vascular alterations and global glomerulosclerosis in this older, predominantly hypertensive cohort were consistent with arterionephrosclerosis, although intermediate stages of glomerular remodeling preceding a focal global glomerulosclerosis pattern were not separately classified in our study.^36^^,^^43^, ^44^, ^45^ The low proportion of globally sclerotic glomeruli may therefore underestimate the extent of arterionephrosclerotic injury.^36^ Importantly, nephron number, glomerular volume and single-nephron eGFR remained associated with impaired compensation after adjustment for age, diabetes, and visceral obesity, supporting their relevance beyond established clinical risk factors. Whether these structural-functional parameters can inform risk-adapted follow-up and therapeutic strategies after nephrectomy merits further investigation.

This study has some limitations. First, patients with dipstick-positive proteinuria were not included to evaluate structural-functional adaptation in the absence of clinically apparent glomerular barrier dysfunction. This exclusion criterion substantially reduced the available sample size and limits the generalizability to individuals without overt proteinuria. Furthermore, the use of dipstick testing to rule out proteinuria might miss some individuals with A2 albuminuria. However, as a retrospective analysis of a real-world cohort, this reflects a common uncertainty in postnephrectomy patient management and emphasizes the clinical relevance of our findings.

Second, key structural parameters were derived using composite stereological approaches. All structural analyses were performed in the resected kidney, which served as a surrogate for the retained contralateral kidney and required the assumption of comparable histomorphometric characteristics between both kidneys. Podocyte counts were derived from podocyte density and glomerular volume and may therefore reflect methodological limitations and measurement variability inherent to stereological approaches. Nevertheless, the inverse relationship between podocyte density and single-nephron filtration, along with increased podocyte nuclear size, is biologically plausible and consistent with podocyte remodeling in response to increased single-nephron workload.

Finally, nephron number estimation and podometrics require ancillary data beyond routine histology, such as computed tomography imaging or additional staining procedures. Although these approaches provide valuable insights, they are labor-intensive and not readily implemented in routine practice. In contrast, NSG density and glomerular volume can be obtained from standard histologic sections without additional radiation exposure or extensive tissue processing, supporting potential clinical applicability.

A core strength of this study is the integration of nephron number, single-nephron filtration, modeled glomerular hemodynamics, and podometrics within the same cohort, whereas most previous studies examined these features in isolation.^6^, ^7^, ^8^, ^9^, ^10^, ^11^, ^12^^,^^17^, ^18^, ^19^, ^20^^,^^24^^,^^37^, ^38^, ^39^^,^^43^ Oncologic nephrectomy additionally provides a human model of abrupt nephron loss with access to large tissue specimens, allowing baseline structural-functional characteristics to be related directly to subsequent functional compensation.

In conclusion, reduced nephron endowment, increased single-nephron filtration, and larger glomerular volume were associated with limited functional recovery during the first year after nephrectomy beyond conventional risk factors. These findings suggest that baseline nephron reserve and filtration workload contribute to interindividual differences in adaptation to abrupt nephron loss. Further studies should determine whether these parameters can guide risk-adapted surveillance and therapeutic strategies aimed at reducing excessive single-nephron workload after nephrectomy.

## Disclosure

All the authors declared no competing interests.

## Supplementary Material

Supplementary File (PDF) **Table S1.** STROBE statement. **Table S2.** Histopathologic scores depending on tertiles of relative ΔeGFR at 1-year follow-up. **Table S3.** Kidney morphologic and histopathologic findings. **Table S4.** Kidney morphologic and histopathologic findings of tertile 3 depending on prevalent type 2 diabetes. **Table S5.** Sensitivity analysis of kidney morphologic and histopathologic parameters depending on tertiles of relative ΔeGFR at 1-year follow-up.

## Footnotes

Supplementary Material (PDF)
**Table S1.** STROBE statement. **Table S2.** Histopathologic scores depending on tertiles of relative ΔeGFR at 1-year follow-up. **Table S3.** Kidney morphologic and histopathologic findings. **Table S4.** Kidney morphologic and histopathologic findings of tertile 3 depending on prevalent type 2 diabetes. **Table S5.** Sensitivity analysis of kidney morphological and histopathological parameters depending on tertiles of relative ΔeGFR at 1-year follow-up.
