# Supplementary material for: Nephron Number, Single-Nephron Workload, and Podometrics as Predictors of Renal Function After Nephrectomy
Source: Kidney Int Rep. 2026 Sep 23;11(12):107099. doi: 10.1016/j.ekir.2026.107099 (PMC13717024; doi:10.1016/j.ekir.2026.107099)
Supplement: Supplementary file 1 [file mmc1.pdf]

Supplementary Material

Supplementary Table S1: STROBE Statement—Checklist of items that should be included in reports of ***cohort studies***

	Item No	Recommendation	Page No
**Title and abstract**	1	(*a*) Indicate the study's design with a commonly used term in the title or the abstract	1
		(*b*) Provide in the abstract an informative and balanced summary of what was done and what was found	1
**Introduction**			
Background/rationale	2	Explain the scientific background and rationale for the investigation being reported	1-2
Objectives	3	State specific objectives, including any prespecified hypotheses	2
**Methods**			
Study design	4	Present key elements of study design early in the paper	2-5
Setting	5	Describe the setting, locations, and relevant dates, including periods of recruitment, exposure, follow-up, and data collection	2-5
Participants	6	(*a*) Give the eligibility criteria, and the sources and methods of selection of participants. Describe methods of follow-up	2
		(*b*) For matched studies, give matching criteria and number of exposed and unexposed	-
Variables	7	Clearly define all outcomes, exposures, predictors, potential confounders, and effect modifiers. Give diagnostic criteria, if applicable	2-5
Data sources/ measurement	8*	For each variable of interest, give sources of data and details of methods of assessment (measurement). Describe comparability of assessment methods if there is more than one group	2-5
Bias	9	Describe any efforts to address potential sources of bias	5
Study size	10	Explain how the study size was arrived at	2 + Fig. 1
Quantitative variables	11	Explain how quantitative variables were handled in the analyses. If applicable, describe which groupings were chosen and why	5
Statistical methods	12	(*a*) Describe all statistical methods, including those used to control for confounding	5
		(*b*) Describe any methods used to examine subgroups and interactions	5
		(*c*) Explain how missing data were addressed	Fig. 1
		(*d*) If applicable, explain how loss to follow-up was addressed	5
		(*e*) Describe any sensitivity analyses	5

**Results**			
Participants	13*	(a) Report numbers of individuals at each stage of study—eg numbers potentially eligible, examined for eligibility, confirmed eligible, included in the study, completing follow-up, and analysed	6 + Fig. 1
		(b) Give reasons for non-participation at each stage	Fig. 1
		(c) Consider use of a flow diagram	Fig. 1
Descriptive data	14*	(a) Give characteristics of study participants (eg demographic, clinical, social) and information on exposures and potential confounders	6-7 + Table 1
		(b) Indicate number of participants with missing data for each variable of interest	
		(c) Summarise follow-up time (eg, average and total amount)	
Outcome data	15*	Report numbers of outcome events or summary measures over time	6-8
Main results	16	(*a*) Give unadjusted estimates and, if applicable, confounder-adjusted estimates and their precision (eg, 95% confidence interval). Make clear which confounders were adjusted for and why they were included	6-8 + Tables 2-3
		(*b*) Report category boundaries when continuous variables were categorized	6-8
		(*c*) If relevant, consider translating estimates of relative risk into absolute risk for a meaningful time period	6-8
Other analyses	17	Report other analyses done—eg analyses of subgroups and interactions, and sensitivity analyses	7-8
**Discussion**			
Key results	18	Summarise key results with reference to study objectives	8-11
Limitations	19	Discuss limitations of the study, taking into account sources of potential bias or imprecision. Discuss both direction and magnitude of any potential bias	11
Interpretation	20	Give a cautious overall interpretation of results considering objectives, limitations, multiplicity of analyses, results from similar studies, and other relevant evidence	8-11
Generalisability	21	Discuss the generalisability (external validity) of the study results	8-11
**Other information**			
Funding	22	Give the source of funding and the role of the funders for the present study and, if applicable, for the original study on which the present article is based	11

*Give information separately for exposed and unexposed groups.

Supplementary Table S2: Histopathological scores depending on tertiles of relative ΔeGFR at 1-year follow up.

	Tertile 1 (n=18)	Tertile 2 (n=18)	Tertile 3 (n=18)	
**Histopathological scores**	**No. (%)**	**No. (%)**	**No. (%)**	***P***
Mesangial expansion				0.72
- none	13 (72%)	12 (67%)	14 (78%)	
- mild	4 (22%)	5 (28%)	4 (22%)	
- intermediate/severe	1 (6%)	1 (6%)	0 (-)	
Intimal thickening/fibrosis				0.60
- none	0 (-)	1 (6%)	0 (-)	
- mild	8 (44%)	4 (22%)	7 (39%)	
- intermediate/severe	10 (56%)	13 (72%)	11 (61%)	
Interstitial fibrosis				0.42
- none	10 (56%)	9 (50%)	13 (72%)	
- mild	5 (28%)	9 (50%)	4 (22%)	
- intermediate/severe	3 (17%)	0 (-%)	1 (6%)	
Tubular atrophy				0.98
- none	11 (61%)	10 (56%)	11 (61%)	
- mild	5 (28%)	8 (44%)	6 (33%)	
- intermediate/severe	2 (11%)	0 (-)	1 (6%)	
Arteriolar hyalinosis				0.12
- none	14 (78%)	16 (89%)	11 (61%)	
- mild	3 (17%)	2 (11%)	4 (22%)	
- intermediate/severe	1 (6%)	0 (-%)	3 (17%)	

Legend: Data were presented as counts and percentages. Percentages were rounded to the next full number. Tertiles 1–3 represent greater, intermediate, and limited compensatory adaptation of the retained kidney after nephrectomy, respectively, based on the relative change in eGFR from baseline to 1-year follow-up (ΔeGFR). Median (interquartile range) ΔeGFR values were -18% (-21 to -6), -33% (-35 to -28), and -48% (-54 to -44) for Tertiles 1, 2, and 3, respectively.

Histopathological alterations were scored semi-quantitatively from between none, mild (°I), intermediate (°II), and severe (°III) lesions.

Kruskal-Wallis test was performed for intergroup comparisons.

Supplementary Table S3: Kidney morphologic and histopathologic findings

**Characteristic**	**n=54**
Kidney morphology	
Parenchymal volume — cm^3	167 (±44)
Cortex volume — cm^3	118 (±33)
Glomerular metrics	
Observed glomerular tufts	300 (±121)
Missing glomerular tufts	13 (4 - 19)
Corrected non-sclerotic glomerular count	312 (±127)
Cortex area — cm^3	116 (±45)
Non-sclerotic glomerular density — per mm^3	16.7 (±5.1)
No. of nephrons per kidney — $\times 10^6$	1.04 (±0.40)
Sclerotic glomeruli — %	1 (0-3)
Glomerular volume — $\times 10^6$ $\mu m^{3\#}$	2.6 (±0.7)
Podometrics	
Podocyte count per glomerulus	514 (±173)
Podocyte density — per 10^6 μm^3	207 (±64)
Apparent podocyte nuclear caliper diameter (*d*) — µm	5.9 (±0.3)
Estimated true podocyte nuclear caliper diameter (*D*) — µm	6.9 (±0.3)
Correction factor for podocyte density estimation	0.403 (±0.014)
Podocyte nuclear volume — μm^3	218 (±32)
Tubular indices	
Tubular count per mm^2 cortical area	190 (±44)
Mean tubular area per mm^2 cortical area — μm^2	5122 (±956)
Nephro-podometrics	
Total glomerular volume per kidney — mm^3	2491 (±1002)
Total podocyte count per kidney — $\times 10^6$	538 (±277)
Filtration and hemodynamic indices	
Single-nephron eGFR — nl/min per $1.73m^2$	45 (±15)
De-indexed single-nephron eGFR — nl/min	50 (±16)
Single-podocyte eGFR — pl/min per $1.73m^2$	98 (±51)
Glomerular capillary pressure — mmHg	55.0 (±6.1)
Oncotic pressure — mmHg	25.6 (±3.5)

Legend:
Gaussian-distributed parameters were displayed
as means and standard deviations (SD),
non-Gaussian distributed data as medians and interquartile ranges.
eGFR – estimated glomerular filtration rate

Supplementary Table S4: Kidney morphologic and histopathologic findings of tertile 3 depending on prevalent type 2 diabetes

**Characteristic**	**Non-T2DM (n=8)**	**T2DM (n=10)**	***P***
Kidney morphology			
Parenchymal volume — cm^3	152 (±52)	153 (±45)	0.96
Cortex volume — cm^3	103 (±40)	107 (±33)	0.82
Glomerular metrics			
No. of nephrons per kidney — $\times 10^6$	0.74 (±0.27)	0.86 (±0.30)	0.39
Non-sclerotic glomerular density — per mm^2	13.8 (±3.0)	15.1 (±3.6)	0.43
Sclerotic glomeruli — %	1 (0-8)	2 (0-4)	0.82
Glomerular volume — $\times 10^6$ $\mu m^{3\#}$	2.9 (±0.7)	3.0 (±0.9)	0.63
Podometrics			
Podocyte count per glomerulus	550 (±153)	576 (±240)	0.79
Podocyte density — per 10^6 μm^3	189 (±64)	197 (±66)	0.80
Podocyte nuclear volume — μm^3	218 (±26)	239 (±34)	0.17
Nephro-podometrics			
Total glomerular volume per kidney — mm^3	2140 (±960)	2396 (±1172)	0.63
Total podocyte count per kidney — $\times 10^6$	411 (±218)	513 (±350)	0.48
Tubular metrics			
Tubular count per mm^2 cortical area	162 (±24)	183 (±34)	0.16
Mean tubular area per mm^2 cortical area — μm^2	5792 (±660)	5183 (±862)	0.12
Filtration and hemodynamic indices			
Single-nephron eGFR — nl/min per $1.73m^2$	57 (±13)	51 (±16)	0.42
De-indexed single-nephron eGFR — nl/min	61 (±13)	58 (±15)	0.62
Single-podocyte eGFR — pl/min per $1.73m^2$	115 (±53)	107 (±63)	0.77
Glomerular capillary pressure — mmHg	56.3 (±5.7)	55.1 (±8.8)	0.75
Oncotic pressure — mmHg	26.1 (±4.0)	23.8 (±2.8)	0.20

Legend:
Gaussian-distributed parameters were displayed
as means and standard deviations (SD),
non-Gaussian distributed data as medians and interquartile ranges.
T2DM – type 2 diabetes mellitus, eGFR – estimated glomerular filtration rate

Supplementary Table S5: Sensitivity analysis of kidney morphological and histopathological parameters depending on tertiles of relative ΔeGFR at 1-year follow up.

**Characteristic**	**Tertile 1 (n=18)**	**Tertile 2 (n=18)**	**Tertile 3 (n=18)**				
	**EMM [95%-CI]**	**EMM [95%-CI]**	**EMM [95%-CI]**	***P***	***P for Age***	***P for eGFR***	***P for VO***
Kidney morphology							
Parenchymal volume — cm³	170 [151-189]	169 [139-200]	148 [125-171]	0.29	0.12	0.29	0.07
Cortex volume — cm³	120 [106-134]	119 [96-142]	103 [86-120]	0.28	0.12	0.31	0.07
Parenchymal volume per m body height — cm³/m	100 [90-110]	97 [81-114]	88 [75-100]	0.31	0.20	0.12	0.08
Cortex volume per m body height — cm³/m	70 [62-78]	69 [56-81]	61 [52-70]	0.30	0.19	0.14	0.08
Parenchymal volume per m² BSA — cm³/m²	91 [82-100]	86 [72-101]	78 [67-89]	0.19	0.41	0.07	0.55
Cortex volume per m² BSA — cm³/m²	64 [57-71]	61 [50-72]	54 [46-62]	0.19	0.37	0.08	0.49
Glomerular metrics							
**No. of nephrons per kidney — ×10⁶**	**1.08 [0.94-1.22]**	**1.26 [1.03-1.48]**	**0.78 [0.62-0.95]** #§	**0.003***	**0.006***	0.08	0.54
**Non-sclerotic glomerular density — per mm³**	**17.0 [14.9-19.2]**	**20.4 [16.9-23.9]**	**14.4 [11.8-17.0]** §	**0.03***	0.36	0.15	0.11
**Glomerular volume — ×10⁶ µm³**#	**2.2 [1.9-2.5]**	**2.2 [1.8-2.7]**	**2.9 [2.6-3.3]** #	**0.009***	0.31	0.68	**0.049***
Podometrics							
Podocyte count per glomerulus	456 [375-536]	502 [373-632]	576 [479-672]	0.17	0.02	0.43	0.65
Podocyte density — per 10⁶ µm³	210 [181-240]	232 [184-280]	200 [164-235]	0.56	0.051	0.45	0.23
Podocyte nuclear volume — µm³	213 [200-226]	203 [182-224]	222 [206-237]	0.37	**0.003***	0.53	**0.03***
Nephro-podometrics							
Total glomerular volume per kidney — mm³	2254 [1901-2607]	2569 [2001-3138]	2208 [1785-2632]	0.56	**0.001***	0.17	**0.008***
Total podocyte count per kidney — ×10⁶	503 [398-607]	639 [470-807]	464 [339-590]	0.25	**<0.001***	0.59	0.41
Tubular metrics							
Tubular count per mm² cortical area	197 [177-218]	181 [148-214]	167 [142-192]	0.18	0.84	0.49	0.42
Mean tubular area per mm² cortical area — µm²	4970 [4520-5419]	5181 [4457-5904]	5648 [5110-6187]	0.16	0.63	0.70	0.57
Filtration and hemodynamic indices							
**Single-nephron eGFR — nl/min per 1.73m²**	**42 [36-49]**	**36 [26-46]**	**58 [50-65]** #§	**0.001***	**0.04***	0.49	0.60
**De-indexed single-nephron eGFR — nl/min**	**46 [39-53]**	**41 [30-52]**	**62 [54-70]** #§	**0.004***	0.06	0.44	0.62
Single-podocyte eGFR — pl/min per 1.73m²	106 [84-129]	78 [42-114]	111 [84-138]	0.32	**0.002***	0.07	0.77

Glomerular capillary pressure — mmHg	55.9 [52.9-58.9]	53.5 [48.6-58.3]	56.4 [52.8-60.0]	0.61	0.81	0.98	0.32
Oncotic pressure — mmHg	25.3 [23.7-26.9]	26.2 [23.6-28.7]	24.8 [22.8-26.7]	0.69	0.003*	0.22	0.48

Legend: Tertiles 1–3 represent greater, intermediate, and limited compensatory adaptation of the retained kidney after nephrectomy, respectively, based on the relative change in eGFR from baseline to 1-year follow-up (ΔeGFR). Median (interquartile range) ΔeGFR values were -18% (-21 to -6), -33% (-35 to -28), and -48% (-54 to -44) for Tertiles 1, 2, and 3, respectively. Parameters were displayed as estimated marginal means (EMM) and 95% confidence intervals (95%-CI).
eGFR – estimated glomerular filtration rate, VO – visceral obesity,
**P*<0.05 in ANCOVA, # *P*<0.05 in Bonferroni-adjusted post hoc analysis vs. tertile 1, § *P*<0.05 in Bonferroni-adjusted post hoc analysis vs. tertile 2.
